# Novel potential causative genes in carotid paragangliomas

**DOI:** 10.1186/s12881-019-0770-6

**Published:** 2019-04-09

**Authors:** Anastasiya V. Snezhkina, Elena N. Lukyanova, Andrew R. Zaretsky, Dmitry V. Kalinin, Anatoly V. Pokrovsky, Alexander L. Golovyuk, George S. Krasnov, Maria S. Fedorova, Elena A. Pudova, Sergey L. Kharitonov, Nataliya V. Melnikova, Boris Y. Alekseev, Marina V. Kiseleva, Andrey D. Kaprin, Alexey A. Dmitriev, Anna V. Kudryavtseva

**Affiliations:** 1 Engelhardt Institute of Molecular Biology, Russian Academy of Sciences, Moscow, Russia; 2 Vishnevsky Institute of Surgery, Ministry of Health of the Russian Federation, Moscow, Russia; 3 National Medical Research Radiological Center, Ministry of Health of the Russian Federation, Moscow, Russia

**Keywords:** Carotid paragangliomas, Tumor-associated genes, Pathogenic variants, High-throughput sequencing, Exome, Transcriptome, Xseq

## Abstract

**Background:**

Carotid paragangliomas (CPGLs) are rare neuroendocrine tumors that arise from the paraganglion at the bifurcation of the carotid artery and are responsible for approximately 65% of all head and neck paragangliomas. CPGLs can occur sporadically or along with different hereditary tumor syndromes. Approximately 30 genes are known to be associated with CPGLs. However, the genetic basis behind the development of these tumors is not fully elucidated, and the molecular mechanisms underlying CPGL pathogenesis remain unclear.

**Methods:**

Whole exome and transcriptome high-throughput sequencing of CPGLs was performed on an Illumina platform. Exome libraries were prepared using a Nextera Rapid Capture Exome Kit (Illumina) and were sequenced under 75 bp paired-end model. For cDNA library preparation, a TruSeq Stranded Total RNA Library Prep Kit with Ribo-Zero Gold (Illumina) was used; transcriptome sequencing was carried out with 100 bp paired-end read length. Obtained data were analyzed using xseq which estimates the influence of mutations on gene expression profiles allowing to identify potential causative genes.

**Results:**

We identified a total of 16 candidate genes (*MYH15, CSP1, MYH3, PTGES3L, CSGALNACT2, NMD3, IFI44, GMCL1, LSP1, PPFIBP2, RBL2, MAGED1, CNIH3, STRA6, SLC6A13,* and *ATM*) whose variants potentially influence their expression (*cis*-effect). The strongest *cis*-effect of loss-of-function variants was found in *MYH15, CSP1,* and *MYH3*, and several likely pathogenic variants in these genes associated with CPGLs were predicted.

**Conclusions:**

Using the xseq probabilistic model, three novel potential causative genes, namely *MYH15, CSP1*, and *MYH3*, were identified in carotid paragangliomas.

## Background

Paragangliomas are rare neuroendocrine neoplasms derived from paraganglionic tissue. In the head and neck, paragangliomas most frequently arise from carotid glomus at the bifurcation of the carotid artery [1]. Carotid paragangliomas (CPGLs) are highly vascularized tumors and are anatomically classified on three groups based on the size and involvement of the carotid artery [2]. The majority of carotid paragangliomas belong to groups 2 and 3 (primarily large size CPGLs with moderate or high arterial attachment), which are surgically challenging. CPGLs are typically characterized by slow-growing and non-aggressive tumors. Up to 10% of CPGLs form regional or distant metastases (malignant CPGLs) [3]. Approximately 10% of CPGLs are hereditary [4]. The new WHO classification describes paragangliomas as tumors with variable metastatic potential [5].

Almost all tumors, including paragangliomas, are characterized by altered energy metabolism [6–8]. Several genes involved in energy metabolism were found to be critical for CPGL pathogenesis. Germline variants in the *SDHB* [9], *SDHC* [10], *SDHD* [11], *RET* [12], and *VHL* [13, 14] genes were associated with carotid paragangliomas. Somatic variants in these genes, as well as ones in *SDHA*, *SDHAF2*, *IDH1*, *NF1, MEN1,* and *KIF1* have also been described in sporadic CPGLs [10, 15, 16].

Germline and somatic variants in *SDHx* genes are often found in head and neck paragangliomas [17, 18]. These genes encode the four subunits (SDHA, SDHB, SDHC, and SDHD) of succinate dehydrogenase (SDH), also known as the mitochondrial complex II. SDH is a component of both the oxidative phosphorylation (OXPHOS) system and the tricarboxylic acid (TCA) cycle, which are key metabolic pathways in the mitochondria. Dysfunctional SDH resulting from genetic or epigenetic alterations can contribute to various pathologies, including cancer [19]. The molecular mechanisms underlying tumorigenesis include the increased generation of reactive oxygen species (ROS) and the disruption of mitochondrial function during apoptosis [20]. On the other hand, dysfunctional SDH leads to succinate accumulation, which in turn affects the activity of the prolyl hydroxylase (PHD) enzyme that catalyzes the hydroxylation of hypoxia inducible factor (HIF). Impaired PHD activity disrupts the binding between HIF and von Hippel-Lindau tumor suppressor protein (pVHL) and subsequently leads to HIF degradation via the ubiquitin-proteasome pathway [21]. The development of paragangliomas is often associated with the pseudohypoxia state caused by the stabilization and activation of HIF [22, 23]. Variants in *SDHD* occur in most carotid paragangliomas, whereas rarer *SDHB* variants result in aggressive disease and metastasis [24–26].

Variants in other known paraganglioma/pheochromocytoma-causative genes (e.g., *RET, NF1,* and *IDH1*) are less common in CPGLs and are primarily observed in sporadic ones. *RET* and *NF1* are a proto-oncogene and a tumor suppressor gene respectively; their protein products participate in the PI3K/AKT and RAS/MAPK pathways and mTOR signaling [27, 28]. Variants in *RET* and *NF1* lead to deregulation of these pathways and are believed to be critical drivers of tumorigenesis. Several variants of *IDH1* but not those of *IDH2* were reported in CPGLs [10, 16]. Inactivation mutations in *IDH1* are known to result in HIF accumulation and tumor development under pseudohypoxia conditions [29]. Two potential pathogenic variants in *KIF1B* were identified in CPGLs [16]. *KIF1B* encodes a motor protein responsible for organelle transport and cell division [30, 31]. Several studies have demonstrated the involvement of KIF1B in tumorigenesis and its tumor suppressor role [32–34]. A study using rat pheochromocytoma cells indicated that the effects of KIF1B in apoptosis are mediated by the EglN3 prolyl hydroxylase pathway [35]. Variants associated with CPGLs were detected in a group of TP53-related genes [*TP53BP1, TP53BP2 (ASPP2),* and *TP53I13 (DSCP1)*] that encode proteins involved in the regulation of cell proliferation, DNA damage response, and apoptosis. These proteins can act as tumor suppressors by modulating p53 function and promoting apoptosis [36–38]. The mutated *ARNT* (*HIF1B*) gene, which encodes an aryl hydrocarbon nuclear translocator, was identified in CPGLs [16]. ARNT functions as a co-factor for various transcription factors, including HIF1A [39]. Aberrant *ARNT* expression can contribute to tumor growth via interactions with specific transcription factors that regulate the expression of multiple genes [40]. In addition, likely pathogenic variants have been identified in *MEN1, BAP1, BRAF, BRCA1, BRCA2, CDKN2A, CSDE1,* and *FGFR3* in individual CPGLs [16]. The above findings indicated high genetic inter-heterogeneity of these tumors.

Although recent studies identified several potential mechanisms involved in paraganglioma pathogenesis, further investigations are required to elucidate the molecular genetic basis of the paragangliomas with different localizations and predispositions to be aggressive. Moreover, most reports focused on mutational status of the genes associated with CPGLs. The functional effects of germline or somatic mutations on gene expression in paragangliomas remain poorly understood. Therefore, the present study aimed to identify the potential causative genes associated with carotid paragangliomas. Using exome data of CPGLs from previous study [16] and RNA-Seq data, we evaluated the effects of somatic variants on gene expression profiles using the xseq probabilistic model. This approach allowed the identification of novel candidate genes and likely pathogenic variants involved in the pathogenesis of CPGLs.

## Methods

### Patients and samples

Carotid paraganglioma samples were collected after obtaining written informed consent from the patients in the Vishnevsky Institute of Surgery. Pathological evaluation was performed for all samples, and the minimum percentage of tumor cells was 80%. The study was approved by the ethics committee from Vishnevsky Institute of Surgery and was conducted in accordance with the principles outlined in the Declaration of Helsinki (1964). Clinicopathologic characteristics of the CPGL patients are presented in Table 1.

**Table 1:** Clinicopathologic characteristics of the CPGL patients

Characteristic	Total, n
Gender	
Male	5
Female	20
Age (years)	
≤ 50	13
> 50	12
Family history of paragangliomas	
Yes	1
No	1
N/A	23

### RNA extraction

Total RNA was extracted from 25 formalin-fixed paraffin-embedded (FFPE) tissues of carotid paragangliomas using a RNeasy FFPE Kit (Qiagen, Germany). RNA concentration was measured on a Qubit 2.0 fluorometer (Thermo Fisher Scientific, USA), and RNA quality was assessed using an Agilent 2100 Bioanalyzer (Agilent Technologies, USA).

### Library preparation and transcriptome sequencing

The cDNA libraries were prepared using a TruSeq Stranded Total RNA Library Prep Kit with Ribo-Zero Gold (Illumina, USA) according to the manufacturer’s instructions. Paired-end (2 × 100 bp) sequencing was performed on an Illumina HiSeq 2500 Sequencing System (Illumina). RNA-Seq data have been deposited to the NCBI Sequence Read Archive (SRA) under accession number PRJNA476709.

Raw reads were subjected to quality control with FastQC (http://www.bioinformatics.babraham.ac.uk/projects/fastqc/). Adapter sequences and low-quality reads were removed using Trimmomatic [41]. Kallisto [42] was used for pseudoalignment and quantifying abundances of transcripts. Gene expression levels were normalized using the TPM (transcripts per million) method.

### Whole-exome sequencing data

We analyzed exome data of carotid paragangliomas generated from a previous study [16]. In that study, exome libraries were prepared using a Nextera Rapid Capture Exome Kit (Illumina). High-throughput sequencing of 75 bp paired-end reads was carried out on a NextSeq500 System (Illumina) at a median sequencing depth of 300×. The raw sequence reads are available at the SRA under the accession number PRJNA411769.

The quality of raw sequencing data was examined with FastQC. Trimmomatic was used to trim low-quality reads and adapter sequences. Reads were then mapped to the reference human genome (GRCh37/hg19) using BWA-MEM [43]. SAMtools software [44, 45] was applied for removing of duplicate reads, sorting, merging, etc. Freebayes [46] was used for variant calling; VCF files were processed with submodules from vcflib (https://github.com/vcflib/vcflib#vcflib). SnpSift from the SnpEff toolbox [47] was used for annotation of genomic variants; the HGMD, OMIM, ClinVar, dbSNP, dbNSFP, COSMIC, ConsensusPathDB, 1000 Genomes Project, and ExAC databases were used as additional resources. Pathogenicity of all variants was assessed in silico using the SIFT [48], PolyPhen2 [49], MutationTaster [50], and LRT [51] algorithms. PhastCons [52] and PhyloP [53] methods were used to analyze the evolutionary conservation of regions harboring the target variants.

Using exome and transcriptome data from the same carotid paragangliomas (25 samples), we evaluated the impact of somatic loss-of-function variants on gene expression profiles using xseq [54]. The xseq model evaluates the posterior probability of the mutation influence in a certain gene on the expression profiles of a number of genes, including itself (*cis-effect*) and associated genes (*trans-effect*). The output of xseq consists of P(F*g,m*) and P(D*g*) values, where P(F*g,m*) is the probability that an individual mutation in gene *g* in an individual patient *m* influences expression within that patient, and P(D*g*) is the probability that a recurrently mutated gene *g* influences gene expression across the population of patients [54].

### Validation of variants by Sanger sequencing

Potential pathogenic variants identified using xseq were selected and validated by Sanger sequencing. Amplification was carried out in 20 μL final reaction volume using a Tersus PCR Kit (Evrogen, Russia). Nested-PCR was performed to amplify the two variants (c.2706-7855G > A and c.2706-7798delA) in the *CSP1* gene. For the first round of amplification, the outer primers (F: 5′-GGAGGCTGAGGCGGGCAGATC-3′ and R1: 5′-CACCATTCTCCTGCCTCAGCCTCT-3′) producing a 181 bp product were used. PCR products from the first step were diluted at 1:1000 and subsequently amplified using the inner primers (F: 5′-GGAGGCTGAGGCGGGCAGATC-3′ and R2: 5′- GTAGCTGGGACTACAGGCGCCT -3′) to produce a 154 bp fragment. PCR and nested-PCR were performed using the following amplification profile: initial denaturation step at 95 °C for 3 min; 35 cycles of denaturation at 95 °C for 30 s, annealing at 60–65 °C for 30 s, and extension at 72 °C for 60 s; and a final extension of 72 °C for 5 min. All PCR products were subjected to 2% agarose gel electrophoresis and visualized using a Gel Doc XR+ Gel Documentation System (BioRad, USA) with GelRed DNA stain (Biotium, USA). Primer sequences are listed in Table 2.

**Table 2:** List of primers used for validation of the variants

Gene	Variant	Primer sequence (5′ → 3′)	Product size (bp)
*CPS1*	c.2706-7855G > A^a^	F: GGAGGCTGAGGCGGGCAGATC R1: CACCATTCTCCTGCCTCAGCCTCT R2: GTAGCTGGGACTACAGGCGCCT	181 154
	c.2706-7798delA^a^		
	c.2706-7745C > G	F1: GAG ACC ATC CTG GCT AAC ACG GTG AAA C R1: GTC TCG CTC TCT CTC CCA GGC TG	195
	p.Gly1382Ser/c.4144G > A	F: CTAAAACTCACACTCACCATAGAAGG R: AGATTCCATGCTGACAGAAAACAAC	276
	p.Arg1748Gln/c.5243G > A	F: TCTCTTCTGCATTTTGACACTCCTG R: TTTCTTTAGAACACAAGCCTCCTCAG	113
*MYH15*	p.Glu1811Lys/c.5431G > A	F: ACTGTCAAGCCCTCATGATTACC R: AAGAACTGAAGAAGAAGCAAGACAC	190
	p.Ala1003Val/c.3008C > T	F: TTCTTCTTAGGTTAAAAACCTTACTGAGG R: GTTTGCTCTTGGTTTTGTTCAAAG	167
*MYH3*	p.Ala1198Thr/c.3592G > A	F: TGACCCGCTGCAGGTTG R: CGTCACCTCCACGCAGATAG	176
	p.Ala1604Thr/c.4810G > A	F: TCAGGTCCCCCTCCATCTTC R: GAGCAGCTGAAGAGGAACTACC	118
	p.Ala1752Thr/c.5254G > A	F: CTCATGCAGCTCCAGAGTGAGG R: GGCTTTAATGCAATGCAATCCCG	141
	p.Ile264Thr/c.791 T > C	F: CAAACACAGGACTCTCTTTCAAACTG R: GCCATGGTAGAATCTGGTCTCC	182
	p.Met798Val/c.2392A > G	F: GAGCTCATGTCTGAACAAAGACCC R: CTTCTGCCCCATAAGGTGTTCTTC	184
	p.Ser1240Leu/c.3719C > T	F: CAGGTTTCAATGACCACGGAGT R: GGAGAAGGAGAAGAGCGAGTTC	142
	p.Thr442Ile/c.1325C > T	F: GATCTGGAACACAAACACAGGACTC R: GGGCCATGGTAGAATCTGGTC	195

^a^Amplification of fragments carrying two variants (c.2706-7855G > A and c.2706-7798delA) in the *CSP1* gene was performed using nested-PCR

## Results

Using the xseq model, we estimated the probability that potential loss-of-function somatic variants (frameshift, nonsense, and splice-site variants) impact gene expression in individual patients [P(F) value] and across the population of patients [P(D) value]. The essential first step was the identification of somatic variants. In the study, we analyzed previously collected data from carotid paraganglioma patients. Tumor samples were collected over a span of more than 20 years in the Vishnevsky Institute of Surgery; no blood samples were obtained from the same patients to date. Thus, data from the 1000 Genomes Project and ExAC databases were used to exclude germline variants. Variants with more than 1% frequency in these databases were excluded from the analysis. We identified 16 genes (*MYH15, CSP1, MYH3, PTGES3L, CSGALNACT2, NMD3, IFI44, GMCL1, LSP1, PPFIBP2, RBL2, MAGED1, CNIH3, STRA6, SLC6A13,* and *ATM*) whose somatic variants can influence their gene expression. These genes had maximum P(F) and P(D) values ranging from 0.51 to 0.99 and from 0.32 to 0.99 respectively. Based on the criteria, *MYH15, CSP1,* and *MYH3* were considered to have significant *cis*-effect of somatic variants on gene expression and were proposed as candidate genes associated with carotid paragangliomas.

To analyze all potential somatic loss-of-function variants in *MYH15, CSP1,* and *MYH3*, novel filter conditions were set for variants derived from the 1000 Genomes Project and ExAC; in particular, variants with frequencies of 2% or less were used in the analysis. Predictions using the SIFT, PolyPhen2, MutationTaster, or LRT algorithms indicated that the *MYH3* gene was predominantly enriched in likely pathogenic variants (Table 3). The PhastCons and PhyloP values for these variants were also high, indicating strong evolutionary conservation. However, several variants were frequently identified in the carotid paraganglioma samples and appear to be germline ones. These variants were excluded from the sample set, and the *cis*-effects were measured again using xseq. In this case, only *MYH15* was characterized by variants that can result in gene expression alterations [average P(F) value – 0.66; and P(D) value – 0.68].

**Table 3:** Likely pathogenic variants in *MYH15, CSP1,* and *MYH3*

Gene	rs ID number	GenBank	Coordinate	Nucleotide change	Amino acid change	Genotype/number of samples	Predictions			
							SIFT	PolyPhen2	MutationTaster	LRT
*CPS1*	–	–	Chr2: 211494571	c.2706-7855G > A	–	Het/2	N/A	N/A	N/A	N/A
	–	–	Chr2: 211494681	c.2706-7745C > G	–	Het/1	N/A	N/A	N/A	N/A
	–	–	Chr2: 211494618	c.2706-7798delA	–	Het/1	N/A	N/A	N/A	N/A
	–	NM_001122633.2	Chr2: 211539650	c.4144G > A	p.Gly1382Ser	Het/2	Tolerated	Benign	Disease-causing	Deleterious
*MYH15*	rs56118396	NM_014981.1	Chr3: 108112954	c.5243G > A	p.Arg1748Gln	Het/4	Tolerated	Benign	Disease-causing	N/A
	–		Chr3: 108110666	c.5431G > A	p.Glu1811Lys	Het/1	Deleterious	Probably damaging	Disease-causing	N/A
*MYH3*	rs34088014	NM_002470.3	Chr17: 10542709	c.3008C > T	p.Ala1003Val	Het/2	Tolerated	Benign	Disease-causing	N/A
	rs61735358		Chr17: 10541497	c.3592G > A	p.Ala1198Thr	Het/1	Tolerated	Possibly damaging	Disease-causing	N/A
	rs201488879		Chr17: 10535939	c.4810G > A	p.Ala1604Thr	Het/1	Deleterious	Possibly damaging	Disease-causing	N/A
	rs34393601		Chr17: 10534960	c.5254G > A	p.Ala1752Thr	Het/3	Deleterious	Possibly damaging	Disease-causing	N/A
	rs763347751		Chr17: 10550688	c.791 T > C	p.Ile264Thr	Het/1	Deleterious	Probably damaging	Disease-causing	N/A
	rs746986821		Chr17: 10543684	c.2392A > G	p.Met798Val	Het/1	Deleterious	Benign	Possibly disease-causing	N/A
	rs551363957		Chr17: 10541370	c.3719C > T	p.Ser1240Leu	Het/1	Deleterious	Benign	Disease-causing	N/A
	rs769788909		Chr17: 10547753	c.1325C > T	p.Thr442Ile	Het/1	Tolerated	Benign	Possibly disease-causing	N/A

SIFT scores range from 0 to 1, and variants with SIFT scores less than 0.05 are considered deleterious. SIFT scores ranging from 0.05 to 1 are predicted to be tolerated variants

The PolyPhen-2 scores range from 0 to 1. Variants with scores ranging from 0 to 0.15 in this range are predicted to be benign. Variants with scores ranging from 0.15 to 0.85 are potential damaging, whereas values above 0.85 have higher probability of being damaging variants

MutationTaster *p*-values (probability) range from 0 to 1. *P*-values close to 1 indicate pathogenicity (disease-causing)

LRT is a likelihood ratio test. LRT scores range from 0 (neutral) to 1 (deleterious)

*Het* heterozygous genotype

All identified variants were validated by Sanger sequencing.

## Discussion

Using integrative analysis of whole exome and RNA-Seq data from a representative sample set, we identified novel potential causative genes in carotid paragangliomas. We employed methods based on the calculation of the posterior probability of a mutation to influence gene expression. From the set of 16 candidate genes, somatic loss-of-function variants in three genes, namely *CPS1, MYH15*, and *MYH3*, were found to significantly alter their gene expression levels.

*CPS1* encodes carbamoyl-phosphate synthase 1, a mitochondrial enzyme that mediates the first step of the urea cycle [55]. Germline variants in *CPS1* cause rare autosomal recessive disorders called carbamoyl-phosphate synthetase I deficiency (CPSID) [56]. Recent studies have indicated that *CPS1* is primarily expressed in liver tissues and participates in the conversion of ammonia to urea in hepatocytes in humans. However, several studies have demonstrated the involvement of CPS1 in carcinogenesis. Alterations in *CPS1* were identified in malignant glioma [57]. CPS1 expression has been associated with the initiation and progression of colorectal cancer [58]. Hypermethylation of *CPS1* accompanied by its downregulated expression was detected in hepatocellular carcinoma [59]. On the other hand, CPS1 overexpression was observed in rectal, lung, and cervical cancers and was correlated with poor prognosis [60, 61]. In lung adenocarcinoma cells, inhibition of CPS1 expression was found to reduce tumor growth [61, 62]. Our current findings revealed that *CPS1* is a potential causative gene in carotid paragangliomas. The variants in *CPS1* can influence its expression level. Both genetic and epigenetic factors influence the expression of *CPS1*, consistent with previous studies and our current findings. Similar to the development of inherited CPSID, variants identified in *CPS1* can be assumed to cause CPS1 inactivation, thereby leading to CPGLs. In turn, *CPS1* variants could lead to alterations in the urea cycle and accumulation of toxic ammonia, which is normally converted to urea. The brain is known to be highly sensitive to the effects of excess ammonia. Ammonia can be transported through the cell membranes in many cell types, including cells of the nervous tissue. In vitro studies have demonstrated that the presence of glutamate and ammonium is necessary for lactate release in astrocytes and glial cells [63]. In turn, extracellular lactate is associated with malignant transformation and can regulate cancer-related signaling pathways (e.g., NF-kB/IL-8, HIF1, and PI3K/Akt/mTOR/Bcl-2), angiogenesis, and ATP production, which in turn influence cell proliferation and migration [64–66]. Such molecular mechanism may be responsible for the development of CPGLs in some cases.

Variants in *MYH3* and *MYH15* were additionally demonstrated to exert *cis*-effects on gene expression in carotid paragangliomas. *MYH3* and *MYH15* belong to the highly conserved myosin heavy chain (MYH) family that encode heavy chains of myosins [67]. Myosins are motor proteins consisting of heavy and light chains and are involved in various types of cellular movements resulting from actin-myosin interactions. In muscle cells, myosin and actin filaments form myofibrils; in non-muscle cells, myosins were demonstrated to participate in various biological processes, such as transport of cellular organelles, chromosomal segregation, actin organization, plasma membrane tension, endocytosis, cell division, and cell motility [68, 69]. Expression of *MYH* genes varies in human tissues. *MYH3* is expressed during the early stages of development in skeletal muscles, and its expression decreases after birth, whereas *MYH15* expression is exclusively detected in adult extraocular muscles [70, 71]. Data from the Human Proteome Atlas (HPA, https://www.proteinatlas.org/) indicate that the peak expression of the MYH3 protein is observed in the heart and skeletal muscles, esophagus, prostate, and seminal vesicle. MYH15 is expressed in the brain, lungs, small intestines, and testes. The Cancer Genome Atlas (TCGA, https://cancergenome.nih.gov/) project, which contains RNA-Seq data from various types of cancer, indicate that *MYH3* and *MYH15* are expressed in almost all tumors at the lowest levels. Slightly elevated mRNA level of *MYH3* gene was found in head and neck cancer with average fragments per kilobase million (FPKM) 3.3 in a set of 499 samples. However, MYH15 proteins are expressed in glioma, urothelial and colorectal cancers (HPA data). In addition, proteome studies revealed deferential expression of MYH15 in early onset dementia, an atypical frontotemporal lobar degeneration (aFTLD) [72].

Variants in these genes were observed in different diseases. Variants in *MYH3* were detected as benzo(a)pyrene exposure-genomic alterations in lung cancer patients [73]. Germline variants in *MYH3* are associated with distal arthrogryposis syndromes [distal arthrogryposis type 1, Freeman-Sheldon syndrome (DA2A), and Sheldon-Hall syndrome (DA2B)], multiple pterygium syndrome (MPS), and spondylocarpotarsal synostosis syndrome (SCT) [74–77]. These disorders are characterized by skeletal anomalies and multiple congenital contractures in the limbs, thereby indicating an essential role of MYH3 in bone development. Variants in *MYH15* were reported to be associated with higher risk for non-cardioembolic stroke and ischemic stroke [78, 79]. In the present study, we identified several likely pathogenic variants in *MYH3* and *MYH15* in carotid paragangliomas, and most of these variants were found to be located in *MYH3*. Currently, the direct roles of MYH3 and MYH15 in normal cellular processes and the development of various pathologies, including cancer, remain unclear. Moreover, only few studies in literature have reported the involvement of MYH3 and MYH15 in tumorigenesis. Therefore, variants identified in *MYH3* and *MYH15* are likely to affect the function of MYH proteins and are potentially associated with carotid paragangliomas. However, further studies are required to elucidate the mechanisms by which MYH3 and MYH15 can promote tumor pathogenesis.

## Conclusions

In the present study, we identified several novel genes, namely *CPS1, MYH15*, and *MYH3*, associated with carotid paragangliomas. These results contribute to a better understanding of the molecular mechanisms behind the pathogenesis of CPGLs.
